# Practical Suggestions Respecting Electricity, Etc., Etc.

**Published:** 1886-04

**Authors:** 


Practical Suggestions Respecting the Varieties of Electric Currents and the Uses of Electricity in Medicine. *By* Ambrose L. Ranney, m.d. *Small octavo, pp. ix, 146, and fourteen plates. New York:* D. Appleton & Co. *Chicago:* Jansen, McClurg & Co.

This little manual contains most of the essential facts regarding the use of electricity in medicine. We can confidently recommend it to those who have not the time to read a larger work, and to undergraduates, who will find it a valuable aid in the clinic and lecture.

That difficult subject, electrophysics, is handled in a brief and creditable manner. The facts given are only those essential to an intelligent application of electricity in medicine.

The personal pronoun of the first person singular occurs with noticeable frequency, and some needless repetitions render the work less readable than it otherwise would be.

The motor points of the extensor aspect of the forearm, in plate iv., are incorrectly drawn.

